# Prognostic Role of Host Cyclooxygenase and Cytokine Genotypes in a Caucasian Cohort of Patients with Gastric Adenocarcinoma

**DOI:** 10.1371/journal.pone.0046179

**Published:** 2012-09-28

**Authors:** María Asunción García-González, David Nicolás-Pérez, Angel Lanas, Luis Bujanda, Patricia Carrera, Rafael Benito, Mark Strunk, Federico Sopeña, Santos Santolaria, Elena Piazuelo, Pilar Jiménez, Rafael Campo, Jesús Espinel, Marisa Manzano, Fernando Geijo, María Pellisé, Ferrán González-Huix, Jorge Espinós, Manuel Zaballa, Llúcia Titó, Luis Barranco, Roberto Pazo, Enrique Quintero

**Affiliations:** 1 Instituto de Investigación Sanitaria Aragón (IIS Aragón), Zaragoza, Spain; 2 Centro de Investigación Biomédica en Red en enfermedades hepáticas y digestivas (CIBERehd), Barcelona, Spain; 3 Department of Gastroenterology, Hospital Universitario de Canarias, Tenerife, Spain; 4 Faculty of Medicine, University of Zaragoza, Zaragoza, Spain; 5 Department of Gastroenterology, Hospital Clínico Universitario, Zaragoza, Spain; 6 Department of Gastroenterology, Hospital Donostia, Faculty of Medicine, University of Basque Country, San Sebastián, Spain; 7 Department of Microbiology, Hospital Clínico Universitario Lozano Blesa, Zaragoza, Spain; 8 Department of Gastroenterology, Hospital San Jorge, Huesca, Spain; 9 Department of Gastroenterology, Hospital Parc Tauli, Sabadell, Spain; 10 Department of Gastroenterology, Complejo Hospitalario de León, León, Spain; 11 Department of Gastroenterology, Hospital 12 de Octubre, Madrid, Spain; 12 Department of Gastroenterology, Hospital Clínico Universitario, Salamanca, Spain; 13 Department of Gastroenterology, Hospital Clinic i Provincial, Barcelona, Spain; 14 Department of Gastroenterology, Hospital Josep Trueta, Girona, Spain; 15 Department of Gastroenterology, Hospital Mútua de Terrassa, Spain; 16 Department of Gastroenterology, Hospital de Cruces, Baracaldo, Spain; 17 Department of Gastroenterology, Hospital de Mataró, Mataró, Spain; 18 Department of Gastroenterology, Hospital del Mar, Barcelona, Spain; 19 Department of Oncology, Hospital Miguel Servet, Zaragoza, Spain; Sun Yat-sen University Medical School, China

## Abstract

**Background:**

Genetic factors influencing the prognosis of gastric adenocarcinoma (GAC) are not well known. Given the relevance of cytokines and other pro-inflammatory mediators in cancer progression and invasiveness, we aimed to assess the prognostic role of several functional cytokine and cyclooxygenase gene polymorphisms in patients with GAC.

**Methodology:**

Genomic DNA from 380 Spanish Caucasian patients with primary GAC was genotyped for 23 polymorphisms in pro-inflammatory (*IL1B*, *TNFA*, *LTA*, *IL6*, *IL12p40*), anti-inflammatory (*IL4*, *IL1RN*, *IL10*, *TGFB1*) cytokine, and cyclooxygenase (*PTGS1* and *PTGS2*) genes by PCR, RFLP and TaqMan assays. Clinical and histological information was collected prospectively. Survival curves were estimated by the Kaplan-Meier method and compared using the log rank test. Outcome was determined by analysis of Cox proportional hazards, adjusting for confounding factors.

**Results:**

The median follow-up period and median overall survival (OS) time were 9.9 months (range 0.4–120.3) and 10.9 months (95% CI: 8.9–14.1), respectively. Multivariate analysis identified tumor stages III (HR, 3.23; 95% CI:2–5.22) and IV (HR, 5.5; 95% CI: 3.51–8.63) as independent factors associated with a significantly reduced OS, whereas surgical treatment (HR: 0.44; 95%CI: 0.3–0.6) was related to a better prognosis of the disease. Concerning genetic factors, none of the 23 polymorphisms evaluated in the current study did influence survival. Moreover, no gene-environment interactions on GAC prognosis were observed.

**Conclusions:**

Our results show that, in our population, the panel of selected pro- and anti-inflammatory cytokine, and cyclooxygenase gene polymorphisms are not relevant in determining the prognosis of gastric adenocarcinoma.

## Introduction

Gastric adenocarcinoma (GAC) still remains the second leading cause of cancer death worldwide. Despite advances in treatment, overall 5-year survival is less than 40% in Western countries [1], [2]. Poor prognosis of patients with GAC has been associated with several conditions such as tumor-node-metastasis (TNM) stage [3], signet-ring cell histology [4], incomplete tumor resection [5], and high lymph node ratio [6]. *Helicobacter pylori* (*H. pylori*) infection and tobacco smoking, two well establish risk factors for gastric carcinogenesis [7]–[9] have been also evaluated as prognostic markers for GAC [10]–[13]. However, few studies have addressed the relevance of patient’s genetic background on the prognosis of the disease. Recently, it has been suggested the correlation between persistence of chronic inflammation and reduced survival in GAC patients [14], [15]. The immune response at the gastrointestinal mucosa is regulated by a wide variety of pro- and anti-inflammatory mediators such as cytokines and growth factors. Among them, interleukin-1β (IL-1β), tumor necrosis factor-α (TNF-α), lymphotoxin-α (LT-α), interleukin-6 (IL-6), and interleukin-12 (IL-12) are potent pro-inflammatory cytokines with a relevant role in both cancer development and progression. Significant high serum levels of IL-1β, TNF-α, and IL-6 have been reported in patients with metastatic GAC [16], [17]. Moreover, upregulation of IL-1β and TNF-α seems to be involved in the molecular mechanisms of the anorexia-cachexia syndrom [18]. By contrast, IL-12 production by PBMCs (peripheral blood mononuclear cells) after stimulation in vitro with tumor cells or LPS (lipopolysaccharide) has been found to be significantly depressed in patients with GAC, specially in the advanced stages of the disease [19].

On the other hand, IL-4, IL-10, and TGF-β1 are potent negative regulators of the Th1-type immune response. Studies carried out in human gastric cancer cell lines found that IL-4 inhibits gastric cell proliferation by blocking cell cycle progression and down-regulating several key G0–G1 cell cycle nuclear factors [20]. IL-10 inhibits macrophage activation, cytokine production, and antigen-specific T-cell proliferation [21]. However, several studies have postulated the involvement of IL-10 in the onset and spread of GAC [22]. Like IL-10, enhanced expression of TGF-β1 has been associated with progression and invasiveness of the GAC [23], [24]. Thus, blockade of TGF-β or TGF-β signaling pathways has been suggested as potential therapy to prevent GAC cells from invading and metastasizing [25].

Besides cytokines and growth factors, cyclooxygenases 1 (COX-1) and 2 (COX-2) have been implicated in carcinogenesis and metastatic progression of many types of cancers including GAC [26], [27]. Cyclooxigenases or prostaglandin G/H synthases (PTGSs) catalyze the formation of prostaglandins from arachidonic acid [28]. COX-1 (PTGS-1) is constitutively expressed and plays a key role in the protection of gastric mucosa. The COX-2 isoform (PTGS-2) is induced in response to cytokines, and other inflammatory and mitogenic stimuli. Whereas the mechanisms by which COX-1 promotes gastric carcinogenesis are not well known, up-regulation of COX-2 has been associated with GAC development and progression by increasing cell proliferation, inhibiting apoptosis, inducing angiogenesis, and suppressing host immune response [29].

Genes encoding the proteins mentioned above harbor polymorphic sites that have been reported to influence transcriptional efficiency and protein levels. At present, there is very little information concerning the influence of immune-related gene variants on the prognosis and clinical outcome of GAC [14], [30]–[32]. Trying to address this specific issue, we aimed to assess the prognostic role of some functional polymorphisms in pro- (*IL1B*, *TNFA*, *LTA*, *IL6, IL12p40*), and anti-inflammatory (*IL1RN*, *IL4, IL10*, *TGFB1*) cytokine as well as in the *PTGS1* and *PTGS2* genes in a large prospective cohort of Spanish Caucasian patients with primary GAC.

## Patients and Methods

### Study Subjects and Data Collection

Consecutive patients diagnosed with primary GAC from May 2002 to December 2003 in 15 general Spanish hospitals were invited to take part in the study. Gastric tumors were grouped according to their anatomical location as cardia GAC (located at the gastroesophageal junction) [33] and non-cardia CGA. Moreover, non-cardia GACs were classified according to the histological type as intestinal, diffuse, or indeterminate [34]. Patients with local recurrence of GAC, non-adenocarcinoma histology, absence of blood samples, or refusal to participate in the study were considered non-eligible. Of the 466 patients with GAC who initially agreed to participate, 400 (85.1%) could be interviewed, had a complete pathology report, and provided biological samples of adequate quality for genetic analysis. However, 20 patients (5%) were excluded from the study due to lack of follow-up. Finally, 380 GAC patients had adequate information to estimate follow-up data and survival analysis. The characteristics of excluded patients did not differ from those of the final group.

At the time of inclusion, detailed information was recorded concerning age, gender, smoking habits, family history of GAC, date of diagnosis, surgical treatment, TNM staging (UICC/AJCC classification), presence of metastases, tumor location, and histological subtype. In addition, approximately 10 ml of peripheral blood from each patient were collected into ethylenediaminetetraacetic acid (EDTA) and serum separator tubes for subsequent DNA extraction and *H. pylori* serology, respectively. Once processed, whole blood and serum samples were aliquoted and stored at –80°C until analysis. All patients gave written informed consent to the study, which was reviewed and approved by the institutional ethics committee of each participating hospital.

### Patient’s Follow-up

Each participant hospital performed the follow-up periodically. Follow-up included computerized tomography of the chest and abdomen, and hematological analysis at 3-month intervals during the first year and thereafter at 6-month intervals. Moreover, an upper digestive endoscopy was performed every year. Information was updated by clinical specialists through in-person interview, medical chart review, and in some cases direct calling. The latest follow-up data in this study were obtained in November 2011.

### Diagnosis of *Helicobacter pylori* Infection

*H. pylori* status was assessed by both urease test (CLO-test; Delta West Ltd., Canning Vale, Bentley, Australia) and histological examination from biopsies taken at the antrum and corpus of the stomach during the endoscopic procedure. The presence of antibodies to CagA and VacA antigens was determined in serum by Western blot analysis (Bioblot Helicobacter; Izasa, Barcelona, Spain), as previously validated in our area [35]. Patients were considered positive for bacterial infection if any of the three tests was positive.

### Genetic Polymorphisms

Genomic DNA was extracted from EDTA-preserved whole blood using the QIAamp DNA Blood Mini extraction kit (Qiagen, Izasa, Barcelona, Spain). We assessed 22 SNP’s (single nucleotide polymorphisms) in the *IL1B*, *TNFA*, *LTA*, *IL12p40*, *IL4*, *IL6, IL10*, *TGFB1*, *PTGS1,* and *PTGS2* genes, as well as the VNTR (variable number of tandem repeat) polymorphism in intron 2 of the *IL1RN* gene. The panel of polymorphisms was selected *a priori* based on three criteria: (a) having a reported prevalence of at least 5% for the less frequent allele among Caucasians; (b) having potential functional consequences leading to altered protein concentrations or protein function; or (c) published evidence of their involvement in GAC development, progression and invasiveness. The regions containing the polymorphic sites were blindly genotyped by RFLP (restriction fragment length polymorphism)-PCR-based methods and TaqMan®-MGB allelic discrimination assays. Quality control for the genotyping was achieved by including a negative PCR control sample (HPLC water) and three positive controls for each SNP analyzed (homozygous for allele 1, heterozygous, and homozygous for allele 2). In addition, 10% of the samples were run twice in separate assays with a genotype concordance of 100% for all the polymorphisms.

#### Cytokine gene analysis

Thirteen SNP’s in the *IL1B* (−511C>T, rs16944 and +3954C>T, rs1143634), *TNFA* (−308G>A, rs1800629 and −238G>A, rs361525), *LTA* (+252G>A, rs909253 and +365G>C, rs746868), *IL12p40* (+1180A>C, rs3212227), *IL4* (−590C>T, rs2243250), *IL6* (+174C>G, rs1800795), *IL10* (−1087G>A, rs1800896 and −597C>A, rs2243250), and *TGFB1* (+869T>C, rs1800470 and +915G>C, rs1800471) genes, as well as the VNTR polymorphism in intron 2 of the *IL-1RN* gene were analysed by PCR-RFLP-based methods and TaqMan®-MGB assays (Applied Biosystems, Madrid, Spain) as previously described [36].

#### PTGS1 genotyping

Subjects were genotyped for three SNPs in the *PTGS1* gene. The *PTGS1*-1676A>G (rs1330344) and +644C>A (rs5788) polymorphisms were analyzed using TaqMan®-MGB pre-designed assays according to the manufacturer’s instructions. The fragment containing the *PTGS1*+50C>T (Pro17Leu) (rs3842787) polymorphic site was amplified as described by Gonzalez-Conejero *et al.* [37]. PCR products were digested with 2 units of *Fau* I (New England Biolabs, Izasa, Barcelona, Spain) for 5 hours at 55°C and electrophoresed on 2% agarose gels. Digests resulted in an intact fragment of 244 bp (allele T) or in two fragments of 125 and 119 bp (allele C).

#### PTGS2 genotyping

Six SNPs located within the *PTGS2* gene were studied. TaqMan® Pre-Designed Assays were used for the detection of the *PTGS2* -1195G>A, (rs689466), +3050 (V102V)G>C, (rs5277), +8473T>C, (rs5275) and +10335G>A, (rs689469) polymorphisms. Genotypes for the *PTGS2*-765G>C (rs20417) and +9850A>G (rs4648298) polymorphisms were determined by PCR-RFLP based methods using primers and reaction profiles as described by Cipollone *et al.* [38] and Cox *et al.* [39], respectively. For typing the rs20417 polymorphism, PCR products were digested with 2 units of *Fau* I (New England Biolabs, Izasa, Barcelona, Spain) for 5 hours at 55°C; digests resulted in two fragments of 122 and 187 bp (allele C) or in an intact fragment of 309 bp (allele G). For typing the rs4648298 polymorphism, PCR products were digested with 2 U of *Alu* I (Invitrogen, Prat de Llobregat, Barcelona, Spain) for 5 h at 37°C. This procedure resulted in 349-bp and 196-bp fragments (allele G) or in the undigested 545-bp fragment (allele A).

### Statistical Analysis

Continuous variables were expressed as mean with standard deviation whereas qualitative variables were expressed as frequencies and percentages. The relationship between qualitative variables was analyzed by contingency tables with chi-square test (χ^2^). Overall survival (OS) time was calculated from the date of the diagnosis to the date of last contact or death from any cause. Patients who where still alive at the last contact and patients lost to follow-up were consider as a censored event in the analysis. In addition, patients’ comorbidity at diagnosis was assessed using a previously validated adaptation of the Charlson Comorbidity Index [40]. Concerning gene polymorphisms, estimated haplotype frequencies and linkage disequilibrium (LD) coefficients (D´and *r^2^*) for the *TGFB1*, *TNFA*, *LTA*, *IL10*, *IL1*, *PTGS1*, and *PTGS2* loci were calculated using the Estimating Haplotype frequencies (EH) software program (available from http://linkage.rockefeller.edu/ott/eh.htm. For each marker, the more common homozygous genotype or haplotype was used as the reference category. Co-dominant and dominant inheritance genetic models were used for analysis. Survival among different genotype groups was estimated using the Kaplan-Meier method and compared using the log rank test. Univariate and multivariate Cox proportional hazards models adjusting for age, gender, *H. pylori* status, smoking habits, tumor location, histological type, tumor stage, treatment, and Charlson index, were performed to evaluate the prognostic value of each polymorphism on patient’s survival. Starting with age and sex, models were constructed using a step-wise forward unconditional method. A variable was entered in the model if the significance level of its coefficient was less than 0.05 and was removed if it was greater than 0.10. Potential interactions between genotypes and clinical and demographic variables were assessed by the corresponding Cox regression models containing the interaction term. For all tests, a two-sided *p*-value <0.05 was considered statistically significant. To address the issue of conducting multiple tests within each polymorphism, an additional Bonferroni correction was applied (*P*-value <0.05/46 = *P*-value <0.001). The statistical analyses were performed using the SPSS software v 15.0 for Windows (SPSS Ibérica, Madrid, Spain).

## Results

### Survival Analysis and Characteristics of GAC Patients

Demographic, clinical, and tumor-related characteristics of patients included in the study are summarized in Table 1. There were 257 males (67.6%) and 123 females (32.4%, male/female ratio 2/1) whose ages ranged from 30 to 96 years. According to Charlson’s index, most of patients (87.6%) had a low morbidity index (<3) at the moment of diagnosis. In 63 cases (16.6%), tumors were located at the cardia, and in 317 cases (83.4%), at the distal region of the estomach. Among distal GACs, 161 (50.8%) were of intestinal histotype, 119 (37.5%) of diffuse histotype, and 37 (11.7%) of mixed or undetermined type.

**Table 1:** Demographic and clinicopathological characteristics of patients with GAC (n = 380).

Variable	Cathegory	TOTAL patients N (%)
Gender	Male	257 (67.6)
	Female	123 (32.4)
Mean age ± SD (yr)		71.2±12
Charlson index	<3 at diagnosis	333 (87.6)
	≥3 at diagnosis	47 (12.4)
Neoplasia location	Proximal	63 (16.6)
	Distal	317 (83.4)
*H. pylori* infection*	Positive	245/344 (71.2)
	Negative	99/344 (28.8)
CagA toxine	Positive	220/344 (64.0)
	Negative	124/344 (36)
VacA	Positive	145/344 (42.2)
	Negative	199/344 (57.8)
Smoking habit	Never	176 (46.3)
	Current	61 (16.1)
	Former	116 (30.5)
	Undetermined	27 (7.1)
TNM stage**	Stage I	55 (14.5)
	Stage II	44 (11.6)
	Stage III	66 (17.4)
	Stage IV	183 (48.2)
	Could not be assesed	32 (8.4)
Curative gastrectomy		170 (44.7)
Chemotherapy		120 (31.6)
Radiotherapy		43 (11.3)
Exitus causes		311 (81.8)
	Neoplasia progression	227 (73)
	Chemotherapy	3 (0.9)
	Surgery	30 (9.6)
	Other causes	51 (16.5)

* Information was available for 344 patients.

** Clinical tumor stages according to the International Union Against Cancer (UICC) criteria.

N = number of individuals.

**Figure 1:**
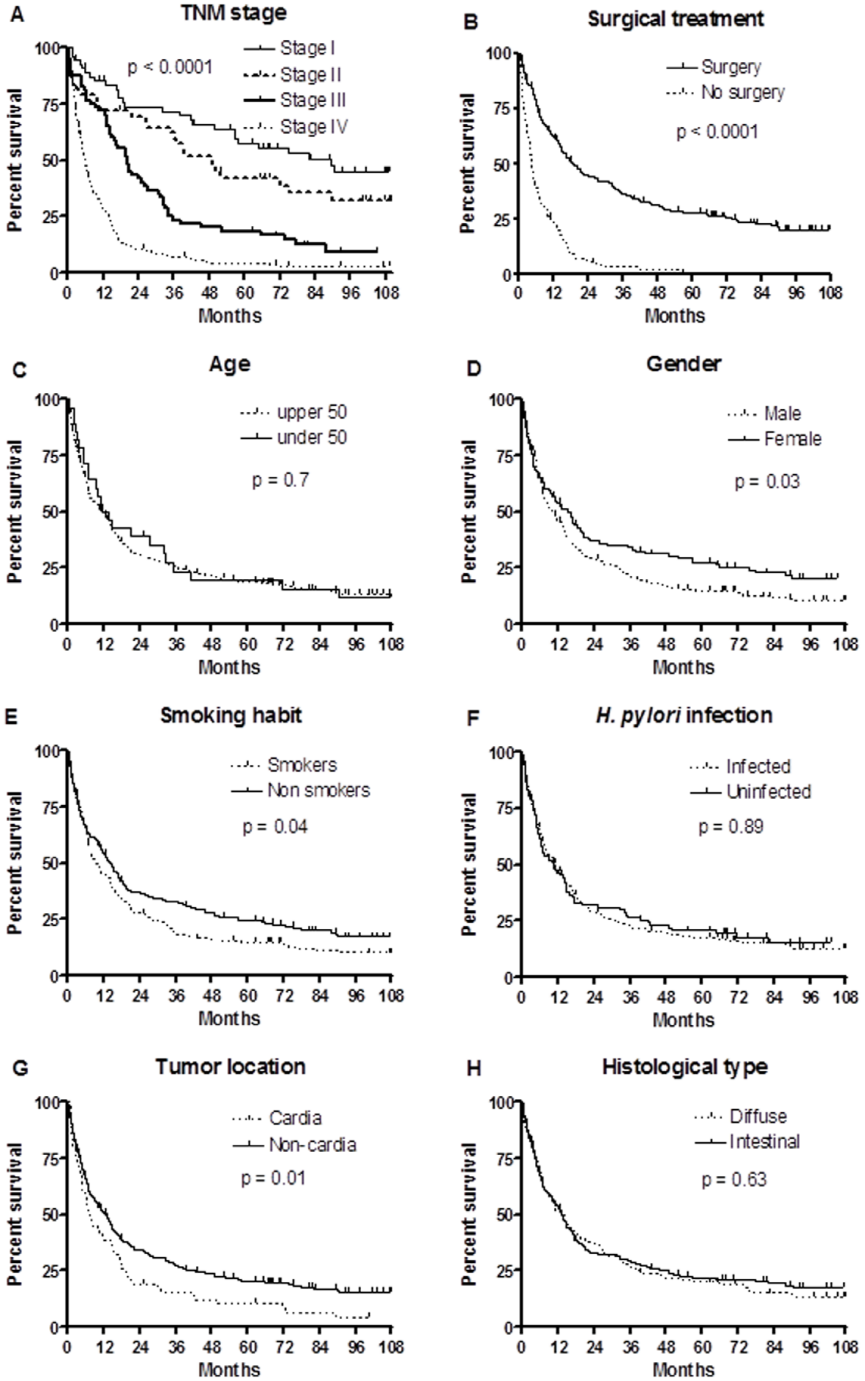
Kaplan-Meier survival plots presented by (A) TNM stage, (B) surgical treatment, (C) age, (D) gender, (E) smoking habit, (F) H. pylori infection, (G) tumor location, and (H) histological subtype. Statistical analysis was performed by the log rank test.

The median follow-up time and the median OS for all patients in our study was 9.9 months (range: 0.4–120.3) and 10.9 months (CI 95%: 8.9–14.1) respectively. The mean and median follow-up for censored patients were 67±42 and 73.4 months (range: 0.6–116.1), respectively. Three hundred and eleven GAC patients (81.8%) had died at the end of the follow-up period, and in 227 cases (73%) death was related to tumor progression. Figure 1 shows Kaplan-Meier survival curves among GAC patients regarding clinicopathological features. In the univariate analysis, male gender, previous or current smoking, proximal location of the tumor, and advanced tumor stages (III and IV) were associated with significantly reduced OS, whereas surgical treatment and a low morbidity index (<3) at the moment of diagnosis were related to a better prognosis of the disease (Table 2). By contrast, other clinicopathological features evaluated in our study such as age, tumor histological type, *H. pylori* status, or treatment with D1 or D2 lymphadenectomy did not influence survival.

**Table 2:** Overall survival analysis according to clinicopathological features.

Variable	Cathegory	N	HR*	95% CI	*P*-value
Gender	Female	123	–	–	
	Male	257	1.31	1.03–1.68	0.031
Age	<50 years	28	–		
	≥50 years	352	1.08	0.72–1.65	0.70
Charlson index	<3 at diagnosis	333	–	–	
	≥3 at diagnosis	47	1.56	1.13–2.17	0.01
Neoplasia location	Non-cardia	317	–	–	
	Cardia	63	1.45	1.09–1.96	0.01
Lauren’s classification	Intestinal	161	–	–	
	Diffuse	119	1.07	0.82–1.39	0.63
*H. pylori* infection	Negative	99	–	–	
	Positive	245	1.02	0.78–1.32	0.89
CagA toxine	Negative	124		–	
	Positive	220	1.07	0.85–1.37	0.57
VacA	Negative	199	–	–	
	Positive	145	0.95	0.75–1.20	0.67
Smoking habit	Never	176	–	–	
	Current and former	177	1.27	1.01–1.61	0.04
TNM stage	Stage I	55	–	–	
	Stage II	44	1.45	0.85–2.46	0.17
	Stage III	66	2.74	1.72–4.36	<0.001
	Stage IV	183	6.30	4.15–9.58	<0.001
Surgical treatment	Yes	247	–	–	
	No	133	3.44	2.68–4.41	<0.001
Lymphadenectomy	D1	57	–	–	
	D2	73	1.13	0.74–1.71	0.58

* Univariate analysis showing unadjusted Hazard Ratio (HR) values.

N = number of individuals.

### Survival Analysis and Gene Polymorphisms

Tables 3 and 4 show the survival hazards ratios among GAC patients according to gene polymorphisms. In the univariate analysis, no significant differences were found when GAC patients were analyzed by genotypes for any of the polymorphisms studied. Only some borderline associations between the *TNFA* rs1800629 (*p* = 0.07 for the AA genotype), *LTA* rs909253 (*p* = 0.06 for the AG genotype), *IL10* rs2243250 (*p* = 0.04 for the CA genotype), and *PTGS2* rs4648298 (*p* = 0.06 for the AG genotype) and OS was observed. Because there were few GAC patients homozygous for the minor alleles of the different polymorphisms, the heterozygous and minor variant homozygous genotypes were combined for additional analysis, assuming a dominant genetic model. Again, no association between OS and any polymorphisms was observed.

**Table 3:** Overall survival analysis according to cytokine gene polymorphisms∧.

Gene	SNP	Genotype	N	HR*	95% CI	*P*-value
*IL1B*	rs16944	CC	178	–	–	–
		CT	164	1.02	0.81–1.29	0.87
		TT	38	1.03	0.70–1.53	0.88
		Carrier T	202	1.02	0.82–1.28	0.85
*IL1B*	rs1143634	CC	222	–	–	–
		CT	139	0.98	0.77–1.24	0.86
		TT	19	1.01	0.61–1.69	0.97
		Carrier T	158	0.98	0.78–1.23	0.88
*TNFA*	rs361525	GG	315	–	–	–
		GA	64	0.78	0.57–1.05	0.1
		AA	1	–	–	–
		Carrier A	65	0.76	0.56–1.02	0.07
*TNFA*	rs1800629	GG	290	–	–	–
		GA	81	1.19	0.91–1.57	0.20
		AA	9	0.40	0.15–1.06	0.07
		Carrier A	90	1.07	0.82–1.40	0.61
*LTA*	rs746868	CC	128	–	–	–
		CG	180	0.94	0.73–1.21	0.64
		GG	72	1.03	0.75–1.42	0.86
		Carrier G	252	1.04	0.82–1.31	0.77
*LTA*	rs909253	AA	222	–	–	–
		AG	121	1.26	0.99–1.61	0.06
		GG	37	1.02	0.68–1.52	0.93
		Carrier G	158	0.83	0.67–1.05	0.12
*IL12B*	rs3212227	AA	232	–	–	–
		AC	126	1.01	0.80–1.29	0.91
		CC	22	1.04	0.65–1.64	0.88
		Carrier C	148	1.02	0.81–1.28	0.88
*IL6*	rs1800795	GG	157	–	–	–
		GC	179	1.01	0.80–1.29	0.92
		CC	44	1.19	0.82–1.73	0.35
		Carrier C	223	1.04	0.83–1.31	0.71
*IL10*	rs2243250	CC	226	–	–	–
		CA	133	0.78	0.62–0.99	0.04
		AA	21	1.07	0.68–1.70	0.77
		Carrier A	154	0.82	0.65–1.03	0.08
*IL10*	rs1800896	AA	112	–	–	–
		GA	195	1.02	0.79–1.32	0.87
		GG	73	1.27	0.92–1.76	0.15
		Carrier G	268	1.08	0.85–1.38	0.53
*TGFB1*	rs1800470	TT	143	–	–	–
		CT	161	0.94	0.73–1.21	0.62
		CC	76	1.21	0.89–1.65	0.23
		Carrier C	237	1.01	0.80–1.27	0.92
*TGFB1*	rs1800471	GG	327	–	–	–
		GC	51	1.06	0.76–1.47	0.73
		CC	2	0.98	0.24–3.96	0.98
		Carrier C	53	0.97	0.76–1.46	0.74
*IL4*	rs2243250	CC	263	–	–	–
		CT	104	0.98	0.76–1.25	0.85
		TT	13	0.95	0.52–1.74	0.86
		Carrier T	117	0.97	0.77–1.24	0.82
*IL1RN*	VNTR**	Carrier allele 2	169	–	–	–
		Non carrier allele 2	211	0.87	0.7–1.19	0.24

∧ A comprehensive analysis was performed for all polymorphisms in the context of different genetic models (dominant, recessive and codominant). Univariate analyses done under codominant and dominant models are shown in the table.

* Unadjusted Hazard Ratio (HR) values.

** Variable number of tandem repeat polymorphism (VNTR) in intron 2 of the *IL1RN* gene. N = number of individuals.

**Table 4:** Overall survival analysis according to *PTGS* gene polymorphisms∧.

Gene	SNP	Genotype	N	HR*	95% CI	*P*-value
*PTGS1*	rs1330344	AA	233	–	–	–
		AG	126	0.85	0.67–1.09	0.20
		GG	21	1.15	0.63–1.83	0.55
		Carrier G	147	0.89	0.71–1.12	0.39
*PTGS1*	rs3842787	CC	342	–	–	–
		CT	36	0.97	0.66–1.41	0.86
		TT	2	1.32	0.33–5.29	0.7
		Carrier T	38	0.98	0.68–1.42	0.93
*PTGS1*	rs5788	CC	283	–	–	–
		CA	88	0.79	0.60–1.03	0.09
		AA	9	1.07	0.53–2.16	0.86
		Carrier A	97	0.81	0.62–1.05	0.11
*PTGS2*	rs689466	AA	234	–	–	–
		AG	136	0.99	0.79–1.25	0.96
		GG	10	0.86	0.41–1.84	0.71
		Carrier G	146	0.99	0.78–1.24	0.90
*PTGS2*	rs20417	GG	252	–	–	–
		GC	116	0.93	0.73–1.19	0.55
		CC	12	1.32	0.74–2.37	0.35
		Carrier C	128	0.96	0.76–1.22	0.74
*PTGS2*	rs5277	GG	253	–	–	–
		GC	116	1.02	0.80–1.30	0.87
		CC	11	0.78	0.39–1.59	0.5
		Carrier C	127	1	0.79–1.26	0.98
*PTGS2*	rs5275	TT	186	–	–	–
		CT	157	0.94	0.74–1.19	0.62
		CC	37	0.95	0.64–1.39	0.78
		Carrier C	194	0.94	0.75–1.18	0.60
*PTGS2*	rs4648298	AA	359	–	–	–
		AG	21	0.62	0.37–1.03	0.06
		GG	0	–	–	–
		Carrier G	21	0.62	0.37–1.03	0.06
*PTGS2*	rs689469	GG	360	–	–	–
		GA	20	0.65	0.39–1.07	0.09
		AA	0	–	–	–
		Carrier A	20	0.65	0.39–1.07	0.09

∧ A comprehensive analysis was performed for all polymorphisms in the context of different genetic models (dominant, recessive and codominant). Univariate analyses done under codominant and dominant models are shown in the table.

* Unadjusted Hazard Ratio (HR) values. N = number of individuals.

Further stratification of patients (Tables S1, S2, and S3 and Figures S1, S2, S3, and S4) by tumor location (cardia vs. non-cardia) and histological subtype (intestinal vs. diffuse) showed no differences in OS among the different gene variants evaluated in the study (Table S4).

An additional haplotype analysis was conducted to evaluate the combined effect of alleles of different polymorphisms on gastric cancer survival. As expected, the *IL1*, *TNFA*, *LTA*, *TGFB1*, *IL10*, *PTGS1*, and *PTGS2* loci were in strong linkage disequilibrium (LD) in our data set (data not shown). Specific LD values for each pair of loci of *IL1*, *TNFA*, *LTA*, *TGFB1*, and *IL10* genes were similar to those reported previously [36]. *PTGS1* and *PTGS2* polymorphisms were also in strong LD with D' values higher than 0.75 with the exception of the *PTGS1*-1676/*PTGS1* 644 loci (D' = 0.21). Table 5 shows the OS analysis according to the most frequent cytokine and *PTGS* haplotypes. As with the independent analysis for each polymorphism, none of the estimated haplotypes showed a significant association with OS.

**Table 5:** Overall survival analysis according to cytokine and *PTGS* estimated haplotypes.

Genes	Haplotypes	N (%)	*HR (95% CI)	*P*-value
***IL1RN/IL1B***	Non-carrier IL1RN*2/IL1B-511 C/IL1B+3954 C	151 (39.7)	0.91 (0.72–1.14)	0.42
	Non-carrier IL1RN*2/IL1B-511 C/IL1B+3954 T	94 (24.7)	0.89 (0.69–1.15)	0.36
	Non-carrier IL1RN*2/IL1B-511 T/IL1B+3954 C	86 (22.6)	0.95 (0.73–1.24)	0.69
	Carrier IL1RN*2/IL1B-511 C/IL1B+3954 C	115 (30.3)	1.17 (0.92–1.48)	0.21
	Carrier IL1RN*2/IL1B-511 C/IL1B+3954 T	57 (15.0)	1.07 (0.78–1.45)	0.68
	Carrier IL1RN*2/IL1B-511 T/IL1B+3954 C	112 (29.5)	1.04 (0.82–1.33)	0.75
	Carrier IL1RN*2/IL1B-511 T/IL1B+3954 T	4 (1.1)	1.25 (0.46–3.34)	0.66
***TNFA/LTA***	TNFA -308 G/TNFA -238 G/LTA Nco I G/LTA Bsi C	88 (23.2)	1.14 (0.91–1.32)	0.62
	TNFA -308 A/TNFA -238 G/LTA Nco I G/LTA Bsi C	91 (23.9)	1.09 (0.84–1.42)	0.52
	TNFA -308 G/TNFA -238 G/LTA Nco I A/LTA Bsi C	154 (40.5)	1.05 (0.84–1.31)	0.67
	TNFA -308 G/TNFA -238 A/LTA Nco I A/LTA Bsi C	65 (17.1)	0.76 (0.56–1.02)	0.07
	TNFA -308 G/TNFA -238 G/LTA Nco I A/LTA Bsi G	252 (66.3)	0.97 (0.76–1.22)	0.76
***TGFB1***	TGFB1+869T/TGFB1+915G	304 (80.0)	0.80 (0.61–1.05)	0.11
	TGFB1+869C/TGFB1+915G	203 (53.4)	1.01 (0.81–1.26)	0.94
	TGFB1+869C/TGFB1+915C	53 (13.9)	1.06 (0.76–1.46)	0.74
***IL10***	IL -10 -597C/IL -10 -1087G	268 (70.5)	1.08 (0.85–1.38)	0.52
	IL -10 -597C/IL -10 -1087A	201 (52.9)	0.94 (0.75–1.17)	0.56
	IL -10 -597A/IL -10 -1087A	154 (40.5)	0.82 (0.65–1.07)	0.09
***PTGS1***	COX1 -1676A/COX1+50C/COX1+644C	351 (92.4)	0.96 (0.64–1.43)	0.82
	COX1 -1676A/COX1+50C/COX1+644A	56 (14.7)	0.72 (0.52–1.05)	0.08
	COX1 -1676G/COX1+50C/COX1+644C	77 (20.3)	0.91 (0.69–1.20)	0.51
	COX1 -1676G/COX1+50C/COX1+644A	45 (11.8)	0.95 (0.68–1.33	0.76
	COX1 -1676G/COX1+50T/COX1+644C	35(9.2)	0.91 (0.62–1.33)	0.62
***PTGS2***	COX2 -1195A/COX2 -765G/COX2+3050G/COX2+8473T/COX2+9850A/COX2+10335G	199 (52.4)	1.13 (0.90–1.41)	0.30
	COX2 -1195A/COX2 -765G/COX2+3050G/COX2+8473C/COX2+9850A/COX2+10335G	85 (22.4)	0.90 (0.69–1.18)	0.44
	COX2 -1195A/COX2 -765G/COX2+3050C/COX2+8473T/COX2+9850A/COX2+10335G	126 (33.2)	1 (0.79–1.27)	0.97
	COX2 -1195A/COX2 -765C/COX2+3050G/COX2+8473C/COX2+9850A/COX2+10335G	107 (28.2)	1.09 (0.85–1.39)	0.51
	COX2 -1195A/COX2 -765C/COX2+3050G/COX2+8473C/COX2+9850G/COX2+10335A	18 (4.7)	0.63 (0.37–1.08)	0.09
	COX2 -1195G/COX2 -765G/COX2+3050G/COX2+8473T/COX2+9850A/COX2+10335G	145 (38.2)	0.92 (0.81–1.27)	0.91
	COX2 -1195A/COX2 -765C/COX2+3050G/COX2+8473T/COX2+9850A/COX2+10335G	5 (1.3)	0.95 (0.35–2.55)	0.91

* Univariate analysis showing unadjusted Hazard Ratio (HR) values. Haplotypes with frequencies lower than 1% are not shown in the table.

N = number of individuals.

Despite the lack of influence of polymorphisms on patient’s survival, we wanted to examine the potential interaction between genotypes and the prognostic factors of GAC identified in our study population (namely TNM stage and surgical treatment). Smoking habit, and *H. pylori* infection, two well known risk factors of GCA were also included for gene-interaction analysis. The exposure variables were as follows: TNM stage (was codified as a continuous variable), surgical treatment (treated vs. untreated), smoking habit (never smokers vs. current smokers), and *H. pylori* infection (positive vs. negative). Tests for interaction under a multiplicative model showed no significant association of any SNPs with tumor stage, surgery, smoking, and *H. pylori* status in relation to survival (Tables S5 and S6). Only a weak significant interaction between the *PTGS2* rs4648298 AA genotype with advance stages and reduced OS was observed (*P*_interaction_ = 0.036).

Gene-gene interactions between all cytokine and *PTGS* polymorphisms were also investigated (Tables S7 and S8). Among all interactions evaluated, the specific interaction between *PTGS1* rs5788 and *TGFB1* rs1800470 gene polymorphisms reached the highest value. Thus, GAC patients carrying both mutant alleles (rs5788A/rs1800470C) had a better OS than non-carriers (median survival 595 days vs. 309; *P*-value = 0.007 by the log rank test; *P*_interaction_ = 0.002). However, after correction for multiple testing, the association did not reach statistical significance (an interaction term *P*-value <0.00001 was considered statistically significant after correction for the number of interactions tested [23×23 = 529; 0.05/529 = 9.5 10^−5^].

In summary, of the environmental and clinicopathologic features evaluated in this study and after controlling for confounding factors, Cox regression analysis identified tumor stages III (HR, 3.23; 95% CI: 2–5.22) and IV (HR, 5.5; 95% CI: 3.51–8.63) as independent factors associated with significantly reduced OS, whereas surgical treatment (HR: 0.44;95%CI: 0.3–0.6) was related to a better prognosis of the disease (Table 6). Concerning genetic factors, none of the polymorphisms evaluated in the current study were related to GAC prognosis. Moreover, no interactions between any SNP’s and the identified prognostic factors were observed.

**Table 6:** Multivariate Cox proportional hazard analysis for GAC patients.

Variable		N*	HR	95% CI	*P*-value
TNM	Stage II	42	1.76	1.01–3.07	0.045
	Stage III	64	3.23	2–5.23	**<0.001**
	Stage IV	176	5.50	3.51–8.62	**<0.001**
Charlson Index ≥3		35	1.55	1.07–2.26	0.02
Surgical treatment		230	0.44	0.32–0.60	**<0.001**

* N = number of individuals. The final number of GAC patients entered in the model after excluding those individuals with missing values was 334 patients. Covariables included in the model were the following: age, gender, Charlson index, smoking habit, neoplasia site, TNM stage, surgical treatment, *TNFA* rs361525, *LTA* rs909253, *IL10* rs2243250, *PTGS1* rs5788, and *PTGS2* rs4648298 gene polymorphisms.

## Discussion

Since the publication in 2000 of the first landmark report by El-Omar and co-workers [41] reporting the association of *IL-1B* rs1143627T and IL1RN*2/*2 genotypes with an increased risk of GAC, numerous studies concerning the association of cytokine gene polymorphisms and GAC risk have been conducted. However, few studies have addressed the role of cytokine gene and other immune mediators on the prognosis of the disease. We analyzed in this study a total of 23 polymorphisms localized in 11 immune-related genes (*IL1B*, *TNFA*, *LTA*, *IL6*, *IL12p40*, *IL1RN*, *IL4, IL10*, *TGFB1*, *PTGS1*, and *PTGS2*). Concerning cytokine genes, none of the polymorphisms analyzed in the current study were related to GAC prognosis. This finding is in contrast with those reported previously in two European studies conducted by Graziano *et al.* [30] and Deans *et al.* [14]. The former [30] reported the association of specific *IL1B and IL1RN* variants (*IL1B* rs16944T/*IL1B* rs1143627C and *IL1RN* 1) with shortened survival of patients with advanced GAC, and the later [14] showed the association of the pro-inflammatory haplotype *IL6* rs1800795C/*IL10* rs1800896G/*TNFA* rs1800629A with an adverse prognosis in patients with squamous esophageal carcinoma or gastroesophageal adenocarcinoma. However, the study by Graziano *et al.* [30] was carried out in a series of 123 GAC patients with recurrent or metastatic tumors treated with palliative chemotherapy, and the study by Deans *et al.* [14] comprised a mixed population of patients with GAC and patients with esophageal squamous cell carcinoma. Our study was performed in a large non-selected cohort of 380 Spanish Caucasian patients with primary GAC in which patients with secondary or recurrent tumors and patients with non-adenocarcinoma histology were excluded at entry. These differences in design and methodology, along with geographical variations in allele frequencies and the plausible effect of other SNPs in these genes could explain, at least in part, the discrepant results reported among studies.

With regard to cyclooxigenases, most studies have implicated COX-2 as the COX isoform involved in cancer development and invasiveness. By contrast, initial evidence for a role of COX-1 in carcinogenesis was scarce. Recent studies reported a high expression of COX-1 in malignant tissues of different types of cancer such as ovarian, breast, and esophageal cancers [42]–[44]. In addition, experimental studies in *Min* mice lacking the *PTGS1* gene showed a reduction in intestinal polyp formation compared to the wild-type *PTGS1*+/*Min* mice [45]. Although the mechanisms by which COX-1 is implicated in carcinogenesis are not well known, it has been suggested that it is mediated through the induction of COX-2 expression via a paracrine mechanism [46], [47]. COX-1 and COX-2 proteins are encoded by the *PTGS1* and *PTGS2* genes which are known to be highly polymorphic. In the last years, several studies have addressed the role of *PTGS1* and *PTGS2* polymorphisms on GAC risk and its precursors with inconsistent results [48]–[53]. However, and to our knowledge, this is the first study evaluating the relevance of *PTGS* variants in the prognosis and survival of GAC patients. In our study none of the *PTGS1* and *PTGS2* polymorphisms were related to GAC prognosis. Only a weak interaction between the rs4648298 polymorphism in the *PTGS2* gene with advance stages of the disease and reduced OS was observed. Recently, two studies by Iglesias *et al* [54] and Coghill *et al* [55] have reported the association of specific *PTGS1* and *PTGS2* gene variants with colorectal cancer survival. The former described a link between the *PTGS2* rs4648298G variant and longer survival in a Spanish population of patients with colorectal cancer. The latter, identified four *PTGS1* polymorphisms influencing colorectal cancer mortality. According to the authors, the rs12132666A variant was associated with 50% lower mortality whereas minor alleles of the rs10306155, rs4836885, and rs5789 resulted in significantly reduced patient’s survival. However, and as point out by the authors, the magnitude of these associations was attenuated after adjustment for stage of the disease at diagnosis. In any case, these studies highlight the need for further research to evaluate whether polymorphisms involved in the prostaglandin synthesis pathway may have the potential to predict survival in patients with GAC.

Similarly to what occurs to cytokine and *PTGS* genes, little information is available with regard to genes encoding growth factors. Among them, *TGFB1* presents special interest due to its role in gastric cancer development, progression and invasiveness [23], [24]. Two functional polymorphisms at positions +869 T>C (rs1800470) and +915 G>C (rs1800471) in the signal protein sequence of the *TGFB1* gene [56], [57] have been related to cancer progression and patient’s survival in several types of cancer [58], [59]. In the current study, neither the rs1800470 nor the rs1800471 polymorphisms were associated with overall survival in GAC patients. Moreover, no interaction with other clinicopathological features such as *H. pylori* status, smoking habit, TNM stage or surgical treatment was observed. In agreement with our results, Guan *et al* [60] found no association between rs1800470 and rs1800471 variants and OS rates, although in this case patients carrying the rs1800471C variant showed a poorer 2-year survival than non carriers.

Besides host factors, we also evaluate the relevance of environmental factors, namely *H. pylori* infection and tobacco smoking, as prognostic markers for GAC. We found that smoking habit was independently associated with a worse prognosis, especially in patients with earlier stages of the disease (HR: 1.78; 95% CI: 1.12–3.5). These findings are in agreement with one of the few studies addressing this issue in which habitual smoking was suggested as an adverse prognostic factor for gastric cancer in Japanese patients [13]. Nicotine is considered the major psychoactive compound of cigarette smoke and it has been well documented to play a key role in gastric cancer [61], [62]. However, its effect on angiogenesis and invasion remains largely unknown, although recent experimental studies have showed that nicotine can stimulate gastric cancer cell proliferation, migration and invasiveness through a COX-2/VEGF dependent pathway [63]. Regarding the influence of *H. pylori* status in GAC outcome, we found no association between bacterial infection and GAC prognosis. Recent studies have suggested that *H. pylori* infection may be related to better prognosis in patients with GAC. Meimarakis *et al.* [64] demonstrated that infection prior to curative-intent resection of GAC correlated with both higher relapse-free and OS rates in early stages cancers (T1 and T2). *H. pylori* status was also found to influence survival in patients with early as well as advanced stages of disease in a subsequent study by Marrelli *et al.* [10]. The reasons for this association are not known but it was explain on the basis of an improved immune response against the tumor induced by *H. pylori* [64]. However, this hypothesis was not confirmed by other studies [65], [66], including our own, suggesting that *H. pylori* negativity may be simply related with more advanced stages or progression of the disease.

Finally, our study has several strengths and limitations. This investigation was carried out in a homogeneous Caucasian population of Spanish patients with primary GAC followed for a long period of time. Moreover, and to our knowledge, this is the first study evaluating the relevance of *PTGS1* and *PTGS2* variants in the prognosis and survival of GAC patients. On the other hand, some limitations should be also considered. In particular, the relatively small sample size limited the power to detect smalls HRs in those low-frequency homozigous variant polymorphisms. Setting an α value of 0.05, the study had a power of 85% to detect HRs >1.4. As a result, it is possible that we could miss minor statistical differences especially when subgroup analyses and assessment of gene-environmental interactions were performed. A second limitation of the study was the lack of a centralized pathological assessment. Evaluation of biopsies and surgical specimens was accomplished at each participant hospital which may represent a source of bias since interobserver variability was not controlled. Variables affecting GAC survival in our study (TNM stage and surgical treatment) have been described as common prognostic factors in the Literature [14], [60], [67]. In the survival analysis, only surgical treatment information was considered for evaluation although data regarding chemo- and radiotherapy were also available. However, radiotherapy and chemotherapy schedules varied considerably among the participating hospitals which precluded a reliable assessment of their effects on disease outcome. Further studies evaluating potencial interactions between clinical and gene polymorphisms on GAC survival should take into consideration the relevance of including a detailed treatment information which could help to identify interactions that may have direct implications for therapy and follow-up strategies.

In summary, our data show that the specific polymorphisms among pro- and anti-inflammatory cytokine and *PTGS* gene polymorphisms evaluated in this study are not related to GAC prognosis in the Spanish population. However, we can not rule out that further studies with larger sample size could detect as statistically significant some small differences found in our study. Future genome-wide association studies (GWAS) and well designed studies in different areas and ethnic groups are needed in order to determine the real contribution of host genetic factors into the prognosis of gastric cancer.

## Supporting Information

Figure S1 **Kaplan-Meier survival plots in cardia GAC patients.** Kaplan-Meier survival plots in cardia GAC patients (n = 63) presented by (A) TNM stage, (B) surgical treatment, (C) age, (D) gender, (E) smoking habit, (F) and *H. pylori* infection status (G). Statistical analysis was performed by the log-rank test.
(TIF)

Figure S2 **Kaplan-Meier survival plots in non-cardia GAC patients.** Kaplan-Meier survival plots in non-cardia GAC patients (n = 317) presented by (A) TNM stage, (B) surgical treatment, (C) age, (D) gender, (E) smoking habit, (F) and *H. pylori* infection status. Statistical analysis was performed by the log-rank test.
(TIF)

Figure S3 **Kaplan-Meier survival plots in intestinal GAC patients.** Kaplan-Meier survival plots in intestinal GAC patients (n = 161) presented by (A) TNM stage, (B) surgical treatment, (C) age, (D) gender, (E) smoking habit, (F) and *H. pylori* infection status. Statistical analysis was performed by the log-rank test.
(TIF)

Figure S4 **Kaplan-Meier survival plots in diffuse GAC patients.** Kaplan-Meier survival plots in diffuse GAC patients (n = 119) presented by (A) TNM stage, (B) surgical treatment, (C) age, (D) gender, (E) smoking habit, (F) and *H. pylori* infection status. Statistical analysis was performed by the log-rank test.
(TIF)

Table S1 **Demographic and clinicopathological characteristics of GAG patients stratified according to the location of the tumor (cardia/non-cardia) and histological subtype (intestinal/diffuse).**
(DOC)

Table S2 **Overall survival analysis and clinicopathological features in GAC patients stratified according to the location of the tumor (cardia/non-cardia).**
(DOC)

Table S3 **Overall survival analysis and clinicopathological features in GAC patients stratified according to the histological type of the tumor (intestinal/diffuse).**
(DOC)

Table S4 **Overall survival analysis and gene polymorphisms according to the location and histological subtype of the tumor.**
(DOC)

Table S5 **Interaction between cytokine gene polymorphisms and clinicopathological features.**
(DOC)

Table S6 **Interaction between** ***PTGS*** **gene polymorphisms and clinicopathological features.**
(DOC)

Table S7 **Gene-gene interactions between cytokine and** ***PTGS*** **gene polymorphisms.**
(DOC)

Table S8 **Gene-gene interactions between cytokine and** ***PTGS*** **gene polymorphisms.**
(DOC)
