# Supplementary figures and images for: Prognostic Role of Host Cyclooxygenase and Cytokine Genotypes in a Caucasian Cohort of Patients with Gastric Adenocarcinoma
Source: PLoS One. 2012 Sep 28;7(9):e46179. doi: 10.1371/journal.pone.0046179 (PMC3460851; doi:10.1371/journal.pone.0046179)

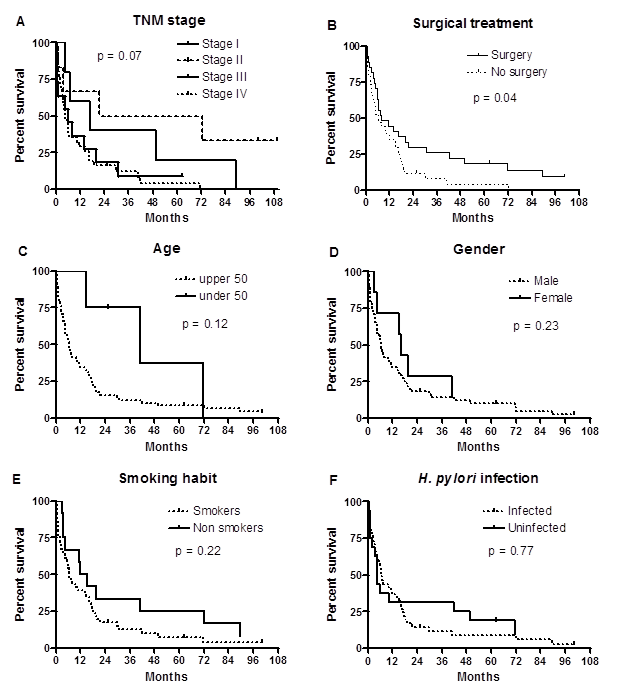

Supplement: Figure S1 — Kaplan-Meier survival plots in cardia GAC patients. Kaplan-Meier survival plots in cardia GAC patients (n = 63) presented by (A) TNM stage, (B) surgical treatment, (C) age, (D) gender, (E) smoking habit, (F) and H. pylori infection status (G). Statistical analysis was performed by the log-rank test. (TIF) [file pone.0046179.s001.tif]

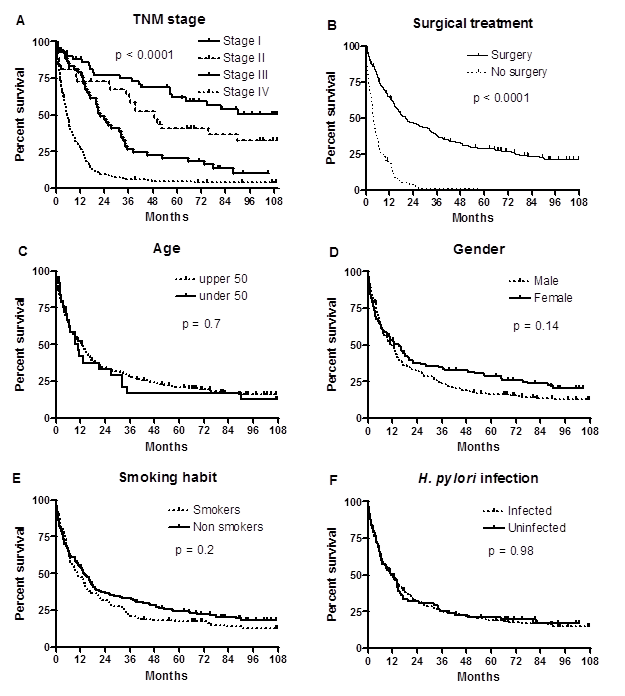

Supplement: Figure S2 — Kaplan-Meier survival plots in non-cardia GAC patients. Kaplan-Meier survival plots in non-cardia GAC patients (n = 317) presented by (A) TNM stage, (B) surgical treatment, (C) age, (D) gender, (E) smoking habit, (F) and H. pylori infection status. Statistical analysis was performed by the log-rank test. (TIF) [file pone.0046179.s002.tif]

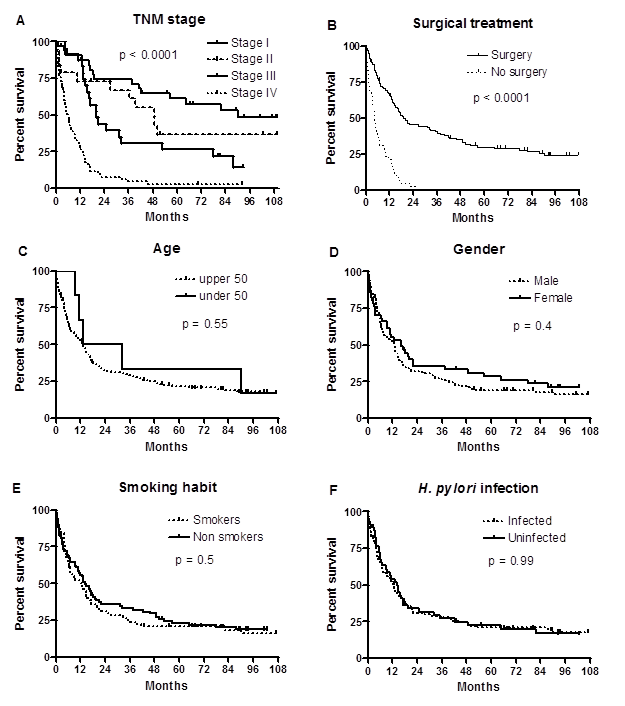

Supplement: Figure S3 — Kaplan-Meier survival plots in intestinal GAC patients. Kaplan-Meier survival plots in intestinal GAC patients (n = 161) presented by (A) TNM stage, (B) surgical treatment, (C) age, (D) gender, (E) smoking habit, (F) and H. pylori infection status. Statistical analysis was performed by the log-rank test. (TIF) [file pone.0046179.s003.tif]

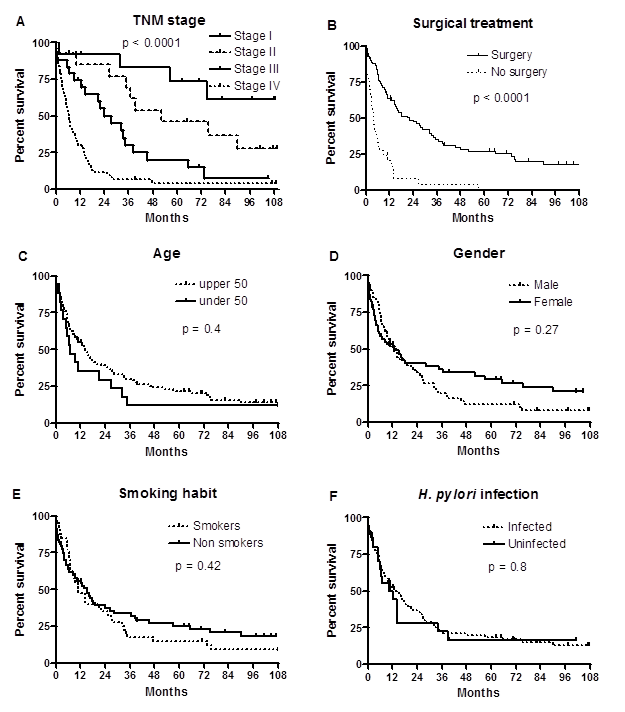

Supplement: Figure S4 — Kaplan-Meier survival plots in diffuse GAC patients. Kaplan-Meier survival plots in diffuse GAC patients (n = 119) presented by (A) TNM stage, (B) surgical treatment, (C) age, (D) gender, (E) smoking habit, (F) and H. pylori infection status. Statistical analysis was performed by the log-rank test. (TIF) [file pone.0046179.s004.tif]
