# Supplementary material for: Prognostic Role of Host Cyclooxygenase and Cytokine Genotypes in a Caucasian Cohort of Patients with Gastric Adenocarcinoma
Source: PLoS One. 2012 Sep 28;7(9):e46179. doi: 10.1371/journal.pone.0046179 (PMC3460851; doi:10.1371/journal.pone.0046179)
Supplement: Table S1 — Demographic and clinicopathological characteristics of GAG patients stratified according to the location of the tumor (cardia/non-cardia) and histological subtype (intestinal/diffuse). (DOC) [file pone.0046179.s005.doc]

**Table S1**. Demographic and clinicopathological characteristics of GAG patients stratified according to the location of the tumor

(cardia/non-cardia) and histological subtype (intestinal/diffuse).

|  |  | **Anatomic location** | | **Histological type** | |
| --- | --- | --- | --- | --- | --- |
| **Variable** | **Cathegory** | **Cardia n= 63** | **Non-cardia n= 317** | **Intestinal n= 161** | **Diffuse n= 119** |
|  |  | **N (%)** | **N (%)** | **N (%)** | **N (%)** |
| Gender | Male | 56 (88.9) | 201 (63.4) | 110 (68.3) | 63 (52.9) |
|  | Female | 7 (11.1) | 116 (36.6) | 51 (31.7) | 56 (47.1) |
| Mean age ± SD (yr) |  | 71.1 ± 11.84 | 70.83 ± 12.37 | 73.4 ± 10.64 | 67.43 ± 13.7 |
| Charlson index | < 3 at diagnosis | 55 (87.3) | 278 (87.7) | 134 (83.2) | 109 (91.6) |
| *H. pylori* infection* | Positive | 41 (71.9) | 204 (71.1) | 99 (67.8) | 84 (74.8) |
| CagA toxine | Positive | 31 (54.4) | 189 (65.9) | 91 (62.3) | 76 (68.8) |
| VacA | Positive | 23 (40.1) | 122 (42.5) | 62 (42.5) | 47 (45.2) |
| Smoking habit | Never | 13 (20.6) | 163 (51.4) | 83 (51.6) | 67 (56.3) |
|  | Current | 15 (23.8) | 46 (14.5) | 21 (13) | 20 (16.8) |
|  | Former | 31 (49.2) | 85 (26.8) | 48 (29.8) | 22 (18.5) |
|  | Undetermined | 4 (6.3) | 23 (7.3) | 9 (5.6) | 10 (8.4) |
| TNM stage** | Stage I | 5 (7.9) | 50 (15.8) | 33 (20.5) | 12 (10.1) |
|  | Stage II | 6 (9.5) | 38 (12) | 19 (11.8) | 14 (11.8) |
|  | Stage III | 11 (17.5) | 55 (17.4) | 24 (14.9) | 25 (21) |
|  | Stage IV | 31 (49.2) | 152 (47.9) | 71 (44.1) | 63 (52.9) |
|  | Could not be assesed | 10 (15.9) | 22 (6.9) | 14 (8.7) | 5 (4.2) |
| Curative gastrectomy |  | 16 (25.4) | 154 (48.6) | 79 (49.1) | 60 (50.4) |
| Chemotherapy |  | 23 (36.5) | 97 (30.6) | 44 (27.3) | 47 (39.5) |
| Radiotherapy |  | 10 (15.9) | 33 (10.4) | 15 (9.3) | 17 (14.3) |
| Exitus causes |  | 59 (93.7) | 252 (79.5) | 128 (79.5) | 94 (79) |
|  | Neoplasia progression | 43 (72.9) | 184 (73) | 95 (74.2) | 67 (71.3) |
|  | Chemotherapy | 1 (1.7) | 2 (0.8) | 0 (0) | 1 (1.1) |
|  | Surgery | 6 (10.2) | 24 (9.5) | 11 (8.6) | 9 (9.6) |
|  | Other causes | 9 (15.2) | 42 (16.7) | 22 (17.2) | 17 (18.1) |

*Information was available for 344 patients.

**Clinical tumor stages according to the International Union Against Cancer (UICC) criteria. N = number of individuals.
