# Supplementary material for: Prognostic Role of Host Cyclooxygenase and Cytokine Genotypes in a Caucasian Cohort of Patients with Gastric Adenocarcinoma
Source: PLoS One. 2012 Sep 28;7(9):e46179. doi: 10.1371/journal.pone.0046179 (PMC3460851; doi:10.1371/journal.pone.0046179)
Supplement: Table S2 — Overall survival analysis and clinicopathological features in GAC patients stratified according to the location of the tumor (cardia/non-cardia). (DOC) [file pone.0046179.s006.doc]

**Table S2**. Overall survival analysis and clinicopathological features in GAC patients stratified according to the location of the tumor (cardia/non-cardia).

|  | **Cardia GAC (n= 63)** | | | **Non-cardia GAC (n= 317)** | | |
| --- | --- | --- | --- | --- | --- | --- |
| **Variable** | **N** | **HR (95% CI)** | ***P*-value** | **N** | **HR (95% CI)** | ***P*-value** |
| Gender |  |  |  |  |  |  |
| Female | 7 | – |  | 116 | – |  |
| Male | 56 | 1.69 (1.72-3.94) | 0.23 | 201 | 1.22 (0.93-1.58) | 0.14 |
| Age |  |  |  |  |  |  |
| < 50 years | 4 | – |  | 24 | – |  |
| ≥ 50 years | 59 | 2.55 (0.79-8.19) | 0.12 | 293 | 0.91 (0.58-1.43) | 0.69 |
| Charlson index |  |  |  |  |  |  |
| < 3 at diagnosis | 55 | – |  | 278 | – |  |
| ≥ 3 at diagnosis | 8 | 1.57 (0.74-3.36) | 0.24 | 39 | 1.56 (1.08-2.24) | 0.02 |
| *H. pylori* infection |  |  |  |  |  |  |
| Negative | 17 | – |  | 82 | – |  |
| Positive | 41 | 1.10 (0.60-2.01) | 0.77 | 204 | 1 (0.75-1.34) | 0.98 |
| CagA |  |  |  |  |  |  |
| Negative | 27 | – |  | 97 | – |  |
| Positive | 31 | 1.11 (0.65-1.91) | 0.7 | 189 | 1.1 (0.83-1.45) | 0.5 |
| VacA |  |  |  |  |  |  |
| Negative | 35 | – |  | 164 | – |  |
| Positive | 23 | 1.28 (0.73-2.23) | 0.39 | 122 | 0.92 (0.71-1.20) | 0.53 |
| Smoking habit |  |  |  |  |  |  |
| Never smoker | 13 | – |  | 163 | – |  |
| Current and former | 46 | 1.51 (0.78-2.91) | 0.22 | 131 | 1.19 (0.92-1.53) | 0.2 |
| TNM stage |  |  |  |  |  |  |
| Stage I | 5 | – |  | 50 | – |  |
| Stage II | 6 | 0.51 (0.13-1.91) | 0.32 | 38 | 1.63 (0.91-2.90) | 0.1 |
| Stage III | 11 | 2.07 (0.67-6.35) | 0.2 | 55 | 2.83 (1.70-4.73) | 0.003 |
| Stage IV | 31 | 6.43 (0.89-6.62) | 0.08 | 152 | 7.36 (4.63-11.7) | <0.001 |
| Surgical treatment |  |  |  |  |  |  |
| No | 35 | – |  | 87 | – |  |
| Yes | 27 | 0.6 (0.34-0.98) | 0.04 | 220 | 0.28 (0.08-0.18) | <0.001 |
| Lymphadenectomy |  |  |  |  |  |  |
| D1 | 1 | – |  | 56 | – |  |
| D2 | 6 | 0.18 (0.01 – 2.93) | 0.2 | 67 | 1.18 (0.76-1.81) | 0.46 |

*Univariate analysis showing unadjusted Hazard Ratio (HR) values.

N = number of individuals.
