# Supplementary material for: Prognostic Role of Host Cyclooxygenase and Cytokine Genotypes in a Caucasian Cohort of Patients with Gastric Adenocarcinoma
Source: PLoS One. 2012 Sep 28;7(9):e46179. doi: 10.1371/journal.pone.0046179 (PMC3460851; doi:10.1371/journal.pone.0046179)
Supplement: Table S3 — Overall survival analysis and clinicopathological features in GAC patients stratified according to the histological type of the tumor (intestinal/diffuse). (DOC) [file pone.0046179.s007.doc]

**Table S3**. Overall survival analysis and clinicopathological features in GAC patients stratified according to the histological type of the tumor (intestinal/diffuse).

|  | **Intestinal GAC (n= 161)** | | | **Diffuse GAC (n= 119)** | | |
| --- | --- | --- | --- | --- | --- | --- |
| **Variable** | **N** | **HR (95% CI)** | ***P*-value** | **N** | **HR (95% CI)** | ***P*-value** |
| Gender |  |  |  |  |  |  |
| Female | 51 | – |  | 56 | – |  |
| Male | 110 | 1.18 (0.8 – 1.73) | 0.4 | 63 | 1.26 (0.84 – 1.91) | 0.27 |
| Age |  |  |  |  |  |  |
| < 50 years | 6 | – |  | 17 | – |  |
| ≥ 50 years | 155 | 1.31 (0.54 – 3.22) | 0.55 | 102 | 0.78 (0.45 – 1.35) | 0.4 |
| Charlson index |  |  |  |  |  |  |
| < 3 at diagnosis | 134 | – |  | 109 | – |  |
| ≥ 3 at diagnosis | 27 | 1.81 (1.16 – 2.82) | 0.009 | 10 | 1.10 (0.51 – 2.38) | 0.81 |
| *H. pylori* infection |  |  |  |  |  |  |
| Negative | 47 | – |  | 20 | – |  |
| Positive | 99 | 1. (0.68 – 1.48) | 0.99 | 84 | 0.93 (0.54 – 1.61) | 0.8 |
| CagA |  |  |  |  |  |  |
| Negative | 55 | – |  | 28 | – |  |
| Positive | 91 | 1.12 (0.77 – 1.63) | 0.57 | 76 | 0.97 (0.60 – 1.57) | 0.89 |
| VacA |  |  |  |  |  |  |
| Negative | 84 | – |  | 57 | – |  |
| Positive | 62 | 1.16 (0.80 – 1.68) | 0.44 | 47 | 1.02 (0.66 – 1.58) | 0.91 |
| Smoking habit |  |  |  |  |  |  |
| Never smoker | 83 | – |  | 67 | – |  |
| Current and former | 69 | 1.13 (0.79 – 1.61) | 0.5 | 42 | 1.21 (0.79 – 1.86) | 0.4 |
| TNM stage |  |  |  |  |  |  |
| Stage I | 33 | – |  | 12 | – |  |
| Stage II | 19 | 1.49 (0.69 – 3.21) | 0.31 | 14 | 2.23 (0.69 – 7.25) | 0.18 |
| Stage III | 24 | 2.35 (1.20 – 4.62) | 0.01 | 25 | 4.58 (1.54 – 13.6) | 0.006 |
| Stage IV | 71 | 7.69 (4.29 – 13.7) | <0.001 | 63 | 10.89 (3.8 – 30.7) | <0.001 |
| Surgical treatment |  |  |  |  |  |  |
| No | 42 | – |  | 34 | – |  |
| Yes | 114 | 0.3 (0.07 – 0.21) | <0.001 | 83 | 0.3 (0.08 – 0.28) | <0.001 |
| Lymphadenectomy |  |  |  |  |  |  |
| D1 | 31 | – |  | 17 | – |  |
| D2 | 30 | 0.90 (0.49 – 1.65) | 0.72 | 31 | 2.01 (0.94 – 4.30) | 0.07 |

*Univariate analysis showing unadjusted Hazard Ratio (HR) values.

N = number of individuals.
