# Supplementary material for: Prognostic Role of Host Cyclooxygenase and Cytokine Genotypes in a Caucasian Cohort of Patients with Gastric Adenocarcinoma
Source: PLoS One. 2012 Sep 28;7(9):e46179. doi: 10.1371/journal.pone.0046179 (PMC3460851; doi:10.1371/journal.pone.0046179)
Supplement: Table S4 — Overall survival analysis and gene polymorphisms according to the location and histological subtype of the tumor. (DOC) [file pone.0046179.s008.doc]

**Table S4**. Overall survival analysis and gene polymorphisms according to the location and histological subtype of the tumor.^

| **SNP ID** | | | **CARDIA GAC** | | | **NON-CARDIA GAC** | | | **INTESTINAL GAC** | | | **DIFFUSE GAC** | | |
| --- | --- | --- | --- | --- | --- | --- | --- | --- | --- | --- | --- | --- | --- | --- |
| Gene | rs number | Change | ref/effect | HR* ( 95% CI) | P | ref/effect | HR* ( 95% CI) | P | ref/effect | HR* ( 95% CI) | P | ref/effect | HR* ( 95% CI) | P |
|  |  |  |  |  | value |  |  | value |  |  | value |  |  | value |
|  |  |  |  |  |  |  |  |  |  |  |  |  |  |  |
| *IL1B* | rs16944 | C > T | 31/32 | 0.81 (0.48 -1.36) | 0.42 | 147/170 | 1.07 (0.84 - 1.38) | 0.57 | 75/86 | 1.22 (0.86 - 1.74) | 0.26 | 59/60 | 0.86 (0.57 - 1.29) | 0.46 |
| *IL1B* | rs1143634 | C > T | 36/27 | 1.33 (0.80 - 2.22) | 0.28 | 186/131 | 0.93 (0.72 - 1.20) | 0.57 | 92/69 | 1.07 (0.75 - 1.52) | 0.71 | 72/47 | 0.78 (0.51 - 1.17) | 0.21 |
| *IL 1RN* | VNTR | Carrier*2/ Non-carrier | 27/36 | 1.06 (0.63 -1.78) | 0.83 | 142/175 | 0.84 (0.65 - 1.08) | 0.27 | 71/90 | 0.68 (0.61 - 1.2) | 0.43 | 51/68 | 1.31 (0.87 - 1.98) | 0.20 |
| *TNFA* | rs361525 | G > A | 51/12 | 0.62 (0.31 - 1.23) | 0.17 | 264/53 | 0.78 (0.56 - 1.09) | 0.15 | 131/30 | 0.73 (0.46 - 1.15) | 0.17 | 101/18 | 0.96 (0.54 - 1.69) | 0.88 |
| *TNFA* | rs1800629 | G > A | 50/13 | 1.41 (0.75 - 2.63) | 0.28 | 240/77 | 1.04 (0.77 - 1.40) | 0.81 | 119/42 | 1.04 (0.69 - 1.56) | 0.86 | 93/26 | 1.1 (0.67 - 1.80) | 0.72 |
| *LTA* | rs746868 | C > G | 14/49 | 0.79 (0.43 -1.44) | 0.43 | 114/203 | 0.96 (0.74 - 1.24) | 0.75 | 55/86 | 1.17 (0.80 - 1.70) | 0.42 | 43/76 | 0.82 (0.54 - 1.24) | 0.35 |
| *LTA* | rs909253 | A > G | 41/22 | 1.34 (0.79 - 2.28) | 0.28 | 181/136 | 1.19 (0.93 - 1.53) | 0.17 | 94/67 | 1.11 (0.78 - 1.59) | 0.55 | 71/48 | 1.14 (0.75 - 1.72) | 0.53 |
| *IL12B* | rs3212227 | A > C | 33/30 | 0.71 (0.42 - 1.21) | 0.21 | 199/118 | 1.05 (0.81 - 1.35) | 0.74 | 101/60 | 0.9 (0.63 – 1.30) | 0.57 | 74/45 | 1.25 (0.82 - 1.88) | 0.3 |
| *IL6* | rs1800795 | G > C | 26/37 | 1.09 (0.65 - 1.84) | 0.74 | 131/186 | 1.04 (0.81 - 1.34) | 0.74 | 64/97 | 1.31 (0.91 - 1.88) | 0.15 | 48/71 | 0.8 (0.53 - 1.21) | 0.3 |
| *IL10* | rs2243250 | C > A | 41/22 | 0.57 (0.32 – 1.06) | 0.08 | 185/132 | 0.88 (0.68 - 1.12) | 0.3 | 98/63 | 0.93 (0.66 - 1.33) | 0.71 | 64/55 | 0.61 (0.40 – 1.08) | 0.07 |
| *IL10* | rs1800896 | A > G | 13/50 | 1.40 (0.74 - 2.65) | 0.3 | 99/218 | 1.02 (0.78 - 1.33) | 0.9 | 48/113 | 1.03 (0.71 - 1.50) | 0.88 | 43/76 | 0.9 (0.59 – 1.37) | 0.63 |
| *TGFB1* | rs1800470 | T > C | 17/46 | 0.86 (0.48 - 1.53) | 0.6 | 126/191 | 1 (0.78 - 1.29) | 1 | 67/94 | 1.01 (0.80 - 1.27) | 0.92 | 41/78 | 0.97 (0.77 – 1.24) | 0.82 |
| *TGFB1* | rs1800471 | G > C | 53/10 | 1.67 (0.84 - 3.32) | 0.15 | 274/43 | 0.96 (0.67 - 1.39) | 0.85 | 142/19 | 0.73 (0.42 - 1.28) | 0.28 | 101/18 | 1.08 (0.60 – 1.94) | 0.8 |
| *IL4* | rs2243250 | C > T | 38/25 | 0.76 (0.44 - 1.30) | 0.31 | 225/92 | 1 (0.75 - 1.29) | 0.92 | 115/46 | 0.95 (0.65 - 1.38) | 0.77 | 85/34 | 1.21 (0.78 – 1.90) | 0.4 |
| *PTGS1* | rs1330344 | A > G | 37/26 | 0.87 (0.51 - 1.47) | 0.59 | 196/121 | 0.89 (0.69 - 1.15) | 0.38 | 103/58 | 0.87 (0.60 - 1.24) | 0.44 | 69/50 | 0.92 (0.61 – 1.38) | 0.67 |
| *PTGS1* | rs3842787 | C > T | 57/6 | 1.09 (0.43 - 2.78) | 0.85 | 285/32 | 0.98 (0.66 - 1.46) | 0.92 | 145/16 | 1.13 (0.65 - 1.96) | 0.68 | 107/12 | 0.86 (0.45 – 1.66) | 0.66 |
| *PTGS1* | rs5788 | C > A | 49/14 | 0.57 (0.29 - 1.10) | 0.1 | 234/83 | 0.87 (0.65 -1.16) | 0.33 | 119/42 | 0.89 (0.60 - 1.22) | 0.56 | 86/33 | 0.82 (0.51 – 1.31) | 0.4 |
| *PTGS2* | rs689466 | A > G | 34/29 | 0.9 (0.54 - 1.51) | 0.69 | 200/117 | 0.97 (0.75 - 1.26) | 0.83 | 98/63 | 0.97 (0.68 - 1.39) | 0.87 | 79/40 | 0.84 (0.54 – 1.29) | 0.42 |
| *PTGS2* | rs20417 | G > C | 44/19 | 0.87 (0.5 – 1.51) | 0.61 | 208/109 | 1 (0.76 - 1.28) | 0.91 | 108/53 | 1.21 (0.84 - 1.75) | 0.3 | 71/48 | 0.9 (0.59 – 1.37) | 0.62 |
| *PTGS2* | rs5277 | G > C | 40/23 | 1.17 (0.69 - 1.99) | 0.56 | 213/104 | 0.96 (0.74 - 1.25) | 0.77 | 108/53 | 0.98 (0.68 - 1.42) | 0.92 | 85/34 | 1.06 (0.68 – 1.67) | 0.79 |
| *PTGS2* | rs5275 | T > C | 28/35 | 0.95 (0.57 - 1.60) | 0.86 | 158/159 | 0.92 (0.72 - 1.18) | 0.53 | 77/84 | 1.09 (0.77 - 1.54) | 0.63 | 54/65 | 0.89 (0.59 – 1.33) | 0.56 |
| *PTGS2* | rs4648298 | A > G | 59/4 | 0.76 (0.27 - 2.11) | 0.6 | 300/17 | 0.58 (0.33-1.05) | 0.08 | 154/7 | 0.47 (0.18 - 1.29) | 0.14 | 110/9 | 0.8 (0.39 – 1.66) | 0.55 |
| *PTGS2* | rs689469 | G > A | 59/4 | 0.76 (0.27 - 2.11) | 0.6 | 301/16 | 0.61 (0.34 - 1.10) | 0.1 | 154/7 | 0.63 (0.26 - 1.55) | 0.31 | 111/8 | 0.72 (0.33 – 1.56) | 0.41 |

^ Univariate analyses done under a dominant model is shown in the table although a comprehensive analysis was performed for all polymorphisms in the context of dominant, recessive and codominant genetic models. *Unadjusted Hazard Ratio (HR) values. Number of patients in the reference category (Ref) and number of patients in the effect category (Effect) assuming a dominat model.
