# Supplementary material for: Prognostic Role of Host Cyclooxygenase and Cytokine Genotypes in a Caucasian Cohort of Patients with Gastric Adenocarcinoma
Source: PLoS One. 2012 Sep 28;7(9):e46179. doi: 10.1371/journal.pone.0046179 (PMC3460851; doi:10.1371/journal.pone.0046179)
Supplement: Table S5 — Interaction between cytokine gene polymorphisms and clinicopathological features. (DOC) [file pone.0046179.s009.doc]

**Table S5**. Interaction between cytokine gene polymorphisms and clinicopathological features.

| **Gene** | **SNP** | **Genotype** | **Smoking^a^** | ***H. pylori*^b^** | **TNM stage^c^** | **Surgery^d^** |
| --- | --- | --- | --- | --- | --- | --- |
|  |  |  |  |  |  |  |
| *IL1B* | rs16944 | CC | 0.526 | 0.665 | 0.870 | 0.644 |
|  |  | CT | 0.707 | 0.850 | 0.713 | 0.658 |
|  |  | TT | 0.258 | 0.372 | 0.794 | 0.510 |
|  |  | Carrier T | 0.490 | 0.668 | 0.850 | 0.868 |
| *IL1B* | rs1143634 | CC | 0.873 | 0.450 | 0.144 | 0.955 |
|  |  | CT | 0.717 | 0.253 | 0.795 | 0.762 |
|  |  | TT | 0.664 | 0.441 | 0.05 | 0.965 |
|  |  | Carrier T | 0.651 | 0.218 | 0.657 | 0.850 |
| *TNFA* | rs361525 | GG | 0.528 | 0.872 | 0.062 | 0.562 |
|  |  | GA | 0.653 | 0.880 | 0.051 | 0.630 |
|  |  | AA | - | 0.880 | - | - |
|  |  | Carrier A | 0.653 | 0.786 | 0.051 | 0.630 |
| *TNFA* | rs1800629 | GG | 0.912 | 0.730 | 0.123 | 0.300 |
|  |  | GA | 0.875 | 0.685 | 0.694 | 0.468 |
|  |  | AA | 0.684 | 0.481 | 0.145 | 0.183 |
|  |  | Carrier A | 0.978 | 0.749 | 0.766 | 0.858 |
| *LTA* | rs746868 | CC | 0.039 | 0.623 | 0.323 | 0.541 |
|  |  | CG | 0.041 | 0.347 | 0.883 | 0.851 |
|  |  | GG | 0.897 | 0.521 | 0.278 | 0.391 |
|  |  | Carrier G | 0.082 | 0.327 | 0.993 | 0.859 |
| *LTA* | rs909253 | AA | 0.320 | 0.813 | 0.803 | 0.779 |
|  |  | AG | 0.255 | 0.683 | 0.584 | 0.484 |
|  |  | GG | 0.474 | 0.560 | 0.728 | 0.962 |
|  |  | Carrier G | 0.570 | 0.599 | 0.792 | 0.538 |
| *IL12B* | rs3212227 | AA | 0.910 | 0.537 | 0.718 | 0.742 |
|  |  | AC | 0.709 | 0.497 | 0.980 | 0.658 |
|  |  | CC | 0.774 | 0.444 | 0.418 | 0.483 |
|  |  | Carrier C | 0.690 | 0.666 | 0.990 | 0.520 |
| *IL6* | rs1800795 | GG | 0.548 | 0.316 | 0.737 | 0.657 |
|  |  | GC | 0.852 | 0.137 | 0.549 | 0.954 |
|  |  | CC | 0.280 | 0.780 | 0.578 | 0.378 |
|  |  | Carrier C | 0.632 | 0.174 | 0.438 | 0.753 |
| *IL10* | rs2243250 | CC | 0.963 | 0.911 | 0.977 | 0.227 |
|  |  | CA | 0.833 | 0.729 | 0.874 | 0.166 |
|  |  | AA | 0.832 | 0.759 | 0.891 | 0.448 |
|  |  | Carrier A | 0.955 | 0.734 | 0.897 | 0.306 |
| *IL10* | rs1800896 | AA | 0.292 | 0.291 | 0.286 | 0.107 |
|  |  | GA | 0.396 | 0.117 | 0.279 | 0.026 |
|  |  | GG | 0.117 | 0.352 | 0.139 | 0.501 |
|  |  | Carrier G | 0.224 | 0.117 | 0.247 | 0.123 |
| *TGFB1* | rs1800470 | TT | 0.604 | 0.625 | 0.715 | 0.640 |
|  |  | CT | 0.324 | 0.346 | 0.492 | 0.621 |
|  |  | CC | 0.542 | 0.847 | 0.754 | 0.347 |
|  |  | Carrier C | 0.285 | 0.464 | 0.501 | 0.492 |
| *TGFB1* | rs1800471 | GG | 0.475 | 0.785 | 0.823 | 0.240 |
|  |  | GC | 0.247 | 0.504 | 0.554 | 0.206 |
|  |  | CC | 0.673 | 0.863 | 0.843 | 0.280 |
|  |  | Carrier C | 0.225 | 0.571 | 0.552 | 0.360 |
| *IL4* | rs2243250 | CC | 0.280 | 0.424 | 0.337 | 0.176 |
|  |  | CT | 0.219 | 0.304 | 0.875 | 0.217 |
|  |  | TT | 0.391 | 0.464 | 0.141 | 0.124 |
|  |  | Carrier T | 0.371 | 0.465 | 0.732 | 0.117 |
| *IL1RN* | VNTR** | Carrier allele 2 | 0.286 | 0.831 | 0.509 | 0.132 |
|  |  | Non carrier allele 2 |  |  |  |  |

*P* interaction values after performing the corresponding Cox regression analyses under a codominant and a dominant genetic models. ^a^Smoking: never vs. current smokers. ^b^*H. pylori* infection status: negative vs. positive. ^c^TNM stage was considered as a continuous variable. ^d^Surgical treatment: treated vs. untreated.
