# Supplementary material for: Prognostic Role of Host Cyclooxygenase and Cytokine Genotypes in a Caucasian Cohort of Patients with Gastric Adenocarcinoma
Source: PLoS One. 2012 Sep 28;7(9):e46179. doi: 10.1371/journal.pone.0046179 (PMC3460851; doi:10.1371/journal.pone.0046179)
Supplement: Table S6 — Interaction between PTGS gene polymorphisms and clinicopathological features. (DOC) [file pone.0046179.s010.doc]

**Table S6**. Interaction between *PTGS* gene polymorphisms and clinicopathological features.

| **Gene** | **SNP** | **Genotype** | **Smoking^a^** | ***H. pylori*^b^** | **TNM stage^c^** | **Surgery^d^** |
| --- | --- | --- | --- | --- | --- | --- |
|  |  |  |  |  |  |  |
| *PTGS1* | rs1330344 | AA | 0.259 | 0.803 | 0.054 | 0.687 |
|  |  | AG | 0.865 | 0.561 | 0.192 | 0.939 |
|  |  | GG | 0.115 | 0.689 | 0.166 | 0.402 |
|  |  | Carrier G | 0.793 | 0.544 | 0.075 | 0.926 |
| *PTGS1* | rs3842787 | CC | 0.364 | 0.087 | 0.082 | 0.704 |
|  |  | CT | 0.364 | 0.053 | 0.082 | 0.704 |
|  |  | TT | - | 0.254 | - | - |
|  |  | Carrier T | 0.303 | 0.134 | 0.080 | 0.710 |
| *PTGS1* | rs5788 | CC | 0.434 | 0.151 | 0.171 | 0.306 |
|  |  | CA | 0.966 | 0.290 | 0.694 | 0.988 |
|  |  | AA | 0.197 | 0.128 | 0.063 | 0.126 |
|  |  | Carrier A | 0.863 | 0.579 | 0.481 | 0.678 |
| *PTGS2* | rs689466 | AA | 0.224 | 0.894 | 0.082 | 0.133 |
|  |  | AG | 0.063 | 0.649 | 0.093 | 0.407 |
|  |  | GG | 0.847 | 0.941 | 0.071 | 0.080 |
|  |  | Carrier G | 0.610 | 0.692 | 0.052 | 0.686 |
| *PTGS2* | rs20417 | GG | 0.135 | 0.762 | 0.591 | 0.680 |
|  |  | GC | 0.328 | 0.761 | 0.537 | 0.391 |
|  |  | CC | 0.107 | 0.479 | 0.413 | 0.768 |
|  |  | Carrier C | 0.638 | 0.721 | 0.521 | 0.415 |
| *PTGS2* | rs5277 | GG | 0.772 | 0.638 | 0.202 | 0.392 |
|  |  | GC | 0.574 | 0.412 | 0.313 | 0.371 |
|  |  | CC | 0.696 | 0.566 | 0.151 | 0.258 |
|  |  | Carrier C | 0.615 | 0.314 | 0.342 | 0.267 |
| *PTGS2* | rs5275 | TT | 0.659 | 0.673 | 0.053 | 0.081 |
|  |  | CT | 0.481 | 0.747 | 0.488 | 0.055 |
|  |  | CC | 0.715 | 0.485 | 0.061 | 0.859 |
|  |  | Carrier C | 0.643 | 0.999 | 0.562 | 0.080 |
| *PTGS2* | rs4648298 | AA |  |  |  |  |
|  |  | AG | 0.208 |  | **0.036** | 0.765 |
|  |  | GG | - | - | - | - |
|  |  | Carrier G | 0.208 |  | **0.036** | 0.765 |
| *PTGS2* | rs689469 | GG |  |  |  |  |
|  |  | GA | 0.433 | 0.189 | 0.065 | 0.683 |
|  |  | AA | - | - | - | - |
|  |  | Carrier A | 0.433 | 0.189 | 0.065 | 0.683 |

*P* interaction values after performing the corresponding Cox regression analyses under a codominant and a dominant genetic models. ^a^Smoking: never vs. current smokers. ^b^*H. pylori* infection status: negative vs. positive. ^c^TNM stage was considered as a continuous variable. ^d^Surgical treatment: treated vs. untreated.
