# Supplementary material for: Prognostic Role of Host Cyclooxygenase and Cytokine Genotypes in a Caucasian Cohort of Patients with Gastric Adenocarcinoma
Source: PLoS One. 2012 Sep 28;7(9):e46179. doi: 10.1371/journal.pone.0046179 (PMC3460851; doi:10.1371/journal.pone.0046179)
Supplement: Table S7 — Gene-gene interactions between cytokine and PTGS gene polymorphisms. (DOC) [file pone.0046179.s011.doc]

**Table S7**. Gene-gene interactions between cytokine and *PTGS* gene polymorphisms.

|  | rs16944 | rs1143634 | rs361525 | rs1800629 | rs746868 | rs909253 | rs3212227 | rs1800795 | *IL1RN* | rs2243250 | rs1800896 | rs1800470 | rs1800471 | rs2243250 |
| --- | --- | --- | --- | --- | --- | --- | --- | --- | --- | --- | --- | --- | --- | --- |
| rs16944 |  | 0.898 | 0.735 | 0.645 | 0.606 | 0.792 | 0.658 | 0.743 | 0.227 | 0.341 | 0.096 | 0.771 | 0.642 | 0.067 |
| rs1143634 | 0.898 |  | 0.907 | 0.165 | 0.765 | 0.019 | 0.163 | 0.780 | 0.946 | 0.433 | 0.731 | 0.254 | 0.918 | 0.034 |
| rs361525 | 0.735 | 0.907 |  | 0.110 | 0.118 | 0.629 | 0.441 | 0.973 | 0.621 | 0.380 | 0.885 | 0.597 | 0.866 | 0.628 |
| rs1800629 | 0.645 | 0.165 | 0.110 |  | 0.175 | . | 0.135 | 0.066 | 0.609 | 0.099 | 0.091 | 0.804 | 0.123 | 0.357 |
| rs746868 | 0.606 | 0.765 | 0.118 | 0.175 |  | 0.169 | 0.236 | 0.492 | 0.957 | 0.325 | 0.552 | 0.355 | 0.559 | 0.200 |
| rs909253 | 0.792 | 0.019 | 0.629 | . | 0.169 |  | 0.078 | 0.091 | 0.939 | 0.648 | 0.497 | 0.644 | 0.284 | 0.740 |
| rs3212227 | 0.658 | 0.163 | 0.441 | 0.135 | 0.236 | 0.078 |  | 0.199 | 0.252 | 0.144 | 0.527 | 0.319 | 0.733 | 0.716 |
| rs1800795 | 0.743 | 0.780 | 0.973 | 0.066 | 0.492 | 0.091 | 0.199 |  | 0.511 | 0.077 | 0.194 | 0.761 | 0.769 | 0.310 |
| *IL1RN* | 0.227 | 0.946 | 0.621 | 0.609 | 0.957 | 0.939 | 0.252 | 0.511 |  | 0.178 | 0.546 | 0.534 | 0.908 | 0.395 |
| rs2243250 | 0.341 | 0.433 | 0.380 | 0.099 | 0.325 | 0.648 | 0.144 | 0.077 | 0.178 |  | 0.711 | 0.711 | 0.809 | 0.036 |
| rs1800896 | 0.096 | 0.731 | 0.885 | 0.091 | 0.552 | 0.497 | 0.527 | 0.194 | 0.546 | 0.711 |  | 0.540 | 0.626 | 0.740 |
| rs1800470 | 0.771 | 0.254 | 0.597 | 0.804 | 0.355 | 0.644 | 0.319 | 0.761 | 0.534 | 0.711 | 0.540 |  | . | 0.131 |
| rs1800471 | 0.642 | 0.918 | 0.866 | 0.123 | 0.559 | 0.284 | 0.733 | 0.769 | 0.908 | 0.809 | 0.626 | . |  | 0.382 |
| rs2243250 | 0.067 | 0.034 | 0.628 | 0.357 | 0.200 | 0.740 | 0.716 | 0.310 | 0.395 | 0.036 | 0.740 | 0.131 | 0.382 |  |
| rs1330344 | 0.121 | 0.336 | 0.907 | 0.192 | 0.494 | 0.875 | 0.530 | 0.770 | 0.914 | 0.425 | 0.251 | 0.937 | 0.788 | 0.315 |
| rs3842787 | 0.221 | 0.905 | 0.637 | 0.958 | 0.959 | 0.446 | 0.472 | 0.253 | 0.149 | 0.184 | 0.783 | 0.154 | 0.387 | 0.491 |
| rs5788 | 0.479 | 0.016 | 0.186 | 0.566 | 0.251 | 0.333 | 0.358 | 0.870 | 0.218 | 0.626 | 0.233 | **0.002** | 0.294 | 0.627 |
| rs689466 | 0.335 | 0.093 | 0.340 | 0.560 | 0.582 | 0.374 | 0.792 | 0.565 | 0.429 | 0.067 | 0.895 | 0.388 | 0.091 | 0.103 |
| rs20417 | 0.055 | 0.038 | 0.740 | 0.755 | 0.311 | 0.899 | 0.752 | 0.636 | 0.144 | 0.922 | 0.252 | 0.662 | 0.624 | 0.706 |
| rs5277 | 0.706 | 0.650 | 0.444 | 0.096 | 0.537 | 0.617 | 0.249 | 0.025 | 0.335 | 0.162 | 0.236 | 0.150 | 0.464 | 0.128 |
| rs5275 | 0.024 | 0.052 | 0.688 | 0.977 | 0.491 | 0.624 | 0.731 | 0.153 | 0.173 | 0.576 | 0.891 | 0.225 | 0.728 | 0.951 |
| rs4648298 | 0.016 | 0.387 | 0.752 | 0.205 | 0.132 | 0.125 | 0.196 | 0.874 | 0.292 | 0.559 | 0.910 | 0.159 | 0.621 | 0.418 |
| rs689469 | 0.055 | 0.315 | 0.816 | 0.894 | 0.052 | 0.184 | 0.430 | 0.738 | 0.218 | 0.662 | 0.526 | 0.119 | 0.701 | 0.157 |

Two-way interactions between all cytokine and *PTGS* polymorphisms studied were investigated. *P* values were obtained after performing Cox regression analysis according to a dominant genetic model. An interaction term *P*-value < 0.00001 was considered statistically significant after correction for the number of interactions tested (23 X 23 = 529; 0.05/529 = 9.5 x 10^-5^).
