# Supplementary material for: Prognostic Role of Host Cyclooxygenase and Cytokine Genotypes in a Caucasian Cohort of Patients with Gastric Adenocarcinoma
Source: PLoS One. 2012 Sep 28;7(9):e46179. doi: 10.1371/journal.pone.0046179 (PMC3460851; doi:10.1371/journal.pone.0046179)
Supplement: Table S8 — Gene-gene interactions between cytokine and PTGS gene polymorphisms. (DOC) [file pone.0046179.s012.doc]

**Table S8**. Gene-gene interactions between cytokine and *PTGS* gene polymorphisms.

|  | rs1330344 | rs3842787 | rs5788 | rs689466 | rs20417 | rs5277 | rs5275 | rs4648298 | rs689469 |
| --- | --- | --- | --- | --- | --- | --- | --- | --- | --- |
| rs16944 | 0.121 | 0.221 | 0.479 | 0.335 | 0.055 | 0.706 | 0.024 | 0.016 | 0.055 |
| rs1143634 | 0.336 | 0.905 | 0.016 | 0.093 | 0.038 | 0.650 | 0.052 | 0.387 | 0.315 |
| rs361525 | 0.907 | 0.637 | 0.186 | 0.340 | 0.740 | 0.444 | 0.688 | 0.752 | 0.816 |
| rs1800629 | 0.192 | 0.958 | 0.566 | 0.560 | 0.755 | 0.096 | 0.977 | 0.205 | 0.894 |
| rs746868 | 0.494 | 0.959 | 0.251 | 0.582 | 0.311 | 0.537 | 0.491 | 0.132 | 0.052 |
| rs909253 | 0.875 | 0.446 | 0.333 | 0.374 | 0.899 | 0.617 | 0.624 | 0.125 | 0.184 |
| rs3212227 | 0.530 | 0.472 | 0.358 | 0.792 | 0.752 | 0.249 | 0.731 | 0.196 | 0.430 |
| rs1800795 | 0.770 | 0.253 | 0.870 | 0.565 | 0.636 | 0.025 | 0.153 | 0.874 | 0.738 |
| *IL1RN* | 0.914 | 0.149 | 0.218 | 0.429 | 0.144 | 0.335 | 0.173 | 0.292 | 0.218 |
| rs2243250 | 0.425 | 0.184 | 0.626 | 0.067 | 0.922 | 0.162 | 0.576 | 0.559 | 0.662 |
| rs1800896 | 0.251 | 0.783 | 0.233 | 0.895 | 0.252 | 0.236 | 0.891 | 0.910 | 0.526 |
| rs1800470 | 0.937 | 0.154 | **0.002** | 0.388 | 0.662 | 0.150 | 0.225 | 0.159 | 0.119 |
| rs1800471 | 0.788 | 0.387 | 0.294 | 0.091 | 0.624 | 0.464 | 0.728 | 0.621 | 0.701 |
| rs2243250 | 0.315 | 0.491 | 0.627 | 0.103 | 0.706 | 0.128 | 0.951 | 0.418 | 0.157 |
| rs1330344 |  | 0.051 | 0.203 | 0.610 | 0.688 | 0.363 | 0.153 | 0.084 | 0.216 |
| rs3842787 | 0.051 |  | 0.641 | 0.648 | 0.064 | 0.390 | 0.466 | 0.893 | 0.943 |
| rs5788 | 0.203 | 0.641 |  | 0.170 | 0.324 | 0.268 | 0.296 | 0.727 | 0.647 |
| rs689466 | 0.610 | 0.648 | 0.170 |  | 0.387 | 0.562 | 0.951 | 0.375 | 0.784 |
| rs20417 | 0.688 | 0.064 | 0.324 | 0.387 |  | 0.531 | 0.885 | 0.553 | 0.657 |
| rs5277 | 0.363 | 0.390 | 0.268 | 0.562 | 0.531 |  | 0.895 | 0.074 | 0.033 |
| rs5275 | 0.153 | 0.466 | 0.296 | 0.951 | 0.885 | 0.895 |  | 0.572 | 0.605 |
| rs4648298 | 0.084 | 0.893 | 0.727 | 0.375 | 0.553 | 0.074 | 0.572 |  | 0.548 |
| rs689469 | 0.216 | 0.943 | 0.647 | 0.784 | 0.657 | 0.033 | 0.605 | 0.548 |  |

Two-way interactions between all cytokine and *PTGS* gene polymorphisms studied were investigated. *P* values were obtained after performing Cox regression analysis according to a dominant genetic model. An interaction term *P*-value < 0.00001 was considered statistically significant after correction for the number of interactions tested (23 X 23 = 529; 0.05/529 = 9.5 x 10^-5^).
